# Neurodegeneration, Mitochondrial Dysfunction, and Oxidative Stress

**DOI:** 10.1155/2013/826046

**Published:** 2013-09-29

**Authors:** Emilio L. Streck, Grzegorz A. Czapski, Cleide Gonçalves da Silva

**Affiliations:** ^1^Laboratory of Bioenergetics, Postgraduate Program in Health Sciences, Health Sciences Unit, University of Southern Santa Catarina, 88806-000 Criciúma, SC, Brazil; ^2^Department of Cellular Signaling, Mossakowski Medical Research Centre, Polish Academy of Sciences, Pawinskiego 5, 02-106 Warsaw, Poland; ^3^Department of Surgery, Beth Israel Deaconess Medical Center, Harvard Medical School, Boston, MA 02215, USA

Mitochondria are intracellular organelles that play a crucial role in energy metabolism. Most cell energy is obtained through mitochondrial metabolic pathways, especially the Krebs cycle and electron transport chain which is the main site for production of reactive oxygen species such as superoxide, hydrogen peroxide, and hydroxyl radicals. Brain tissue is highly sensitive to oxidative stress due to its high oxygen consumption, iron and lipid contents, and low activity of antioxidant defenses. Thus, energy metabolism impairment and oxidative stress are important events that have been related to the pathogenesis of diseases affecting the central nervous system.

In the present issue, the pathogenesis of common Alzheimer's (AD) and Parkinson's (PD) diseases is addressed in five papers. Mondragón-Rodríguez et al. proposed that phosphorylated tau protein could play the role of potential connector and that a combined therapy involving antioxidants and check points for synaptic plasticity during early stages of the disease could become a viable therapeutic option for AD treatment. This paper is accompanied by a study by T. Rohn that explores the potential role that the triggering receptor expressed on myeloid cells 2 (TREM2) normally plays and how loss of function may contribute to AD pathogenesis by enhancing oxidative stress and inflammation within the central nervous system. Additionally, O. Myhre et al. review the possible impact of environmental exposures in metal dyshomeostasis and inflammation in AD and PD. Furthermore, T. Omura et al. explore recent studies on the mechanism of endoplasmic reticulum stress-induced neuronal death related to PD, focusing on the involvement of human ubiquitin ligase HRD1 in the prevention of neuronal death as well as a potential therapeutic approach for PD based on the upregulation of HRD1. Lastly, S. Matsuda et al. showed a concise overview on the cellular functions of the mitochondrial kinase PINK1 and the relationship between Parkinsonism and mitochondrial dynamics, with particular emphasis on a mitochondrial damage response pathway and mitochondrial quality control.

Three of the papers deal with aspects of oxidative stress implicated in the pathogenesis of neurodegenerative diseases. W. Liu et al. reviewed the current literature on the effects of oxidative stress due to exhaustive training on uncoupling protein 2 (UCP2) and Bcl-2/Bax in rat skeletal muscles. A.-M. Enciu et al. explore the possibility of oxidative-induced molecular mechanisms of blood-brain barrier disruption and tight junction protein expression alteration, in relation to aging and neurodegeneration. Moreover, M. Tajes et al. suggest that peroxynitrite induces cell death and is a very harmful agent in brain ischemia.

A. Hosseini and M. Abdollahi review the pathogenesis of diabetic neuropathy with a focus on oxidative stress and introduced therapies dependent or independent of oxidative stress. A. Sekigawa et al. review the currently available evidence that neither mitochondria nor leucine-rich repeat kinase 2 (LRRK2) was present in the swellings of mice expressing P123H *β*-synuclein, suggesting that *α*- and *β*-synucleins might play differential roles in the mitochondrial pathology of *α*-synucleinopathies. The paper by P. F. Schuck et al. showed that *trans*-glutaconic acid is toxic to brain cells *in vitro*, by causing alterations in cell ion balance and probably neurotransmission, as well as oxidative stress in rat cerebral cortex. To complete the issue, M. J. Rodríguez et al. discuss the mitochondrial K_ATP_ channel as a new target to control microglia activity, avoid its toxic phenotype, and facilitate a positive disease outcome. Fortes et al. showed that 5TIO1 can protect the brain against neuronal damage regularly observed during neuropathologies. These papers are accompanied by a review by Z. Yu et al. on how the neuroglobin's neuroprotection is related to mitochondria function and regulation. 

By compiling these papers, we hope to enrich our readers and researchers with respect to mitochondrial dysfunction, energy metabolism impairment, and oxidative stress in the pathophysiology of neurodegenerative diseases.

*Emilio L. Streck* *Emilio L. Streck* *Grzegorz A. Czapski* *Grzegorz A. Czapski* *Cleide Gonçalves da Silva* *Cleide Gonçalves da Silva*

